# Supplementary material for: Content-rich biological network constructed by mining PubMed abstracts
Source: BMC Bioinformatics. 2004 Oct 8;5:147. doi: 10.1186/1471-2105-5-147 (PMC528731; doi:10.1186/1471-2105-5-147)
Supplement: Additional File 2 — The original results of the above study (non-essential files are deleted to keep the file size under the limit set by BMC bioinformatics). [file 1471-2105-5-147-S2.bz2 › chilibotAdditionalFile2/dip10/705ID10734142E18042/html/MUTH_MUTS.html]

 


 **MUTH** and **MUTS** 
  
Found 88 abstracts in PubMed, retrieved 10.  
 

 What does Google say? 
 PDF only 
| .edu only 

---

**Interactive relationship** (e.g. stimulation, inhibition, etc)

**Neutral relationship**- **MutS**  initiates repair by binding to the mismatch, and activates together with MutL the  **MutH**  endonuclease, which incises at hemimethylated dam sites and thereby mediates strand discrimination.  Ref: 11920679 J Cell Physiol, 2002

**Non-interactive relationship** (e.g. studied together, co-existance, homology, etc.)

- Alteration of residues in either the mismatch recognition domain, the ATPase active site, or the domain interfaces linking the two activities can diminish the differential binding of  **MutS**  to homoduplex versus heteroduplex and results in the loss of mismatch specific  **MutH**  activation.  Ref: 12606120 DNA Repair Amst , 2003
- As compared to native  **MutS** , the ability of MutS800 to promote MutL dependent activation of  **MutH**  is substantially reduced.  Ref: 12829697 J Biol Chem< MedlineTA>J Biol Chem, 2003
- Whereas  **mutS**  and mutL genes are found in most organisms, the  **mutH**  gene is limited to some proteobacteria.  Ref: 11934505 FEMS Microbiol Lett, 2002
- We have used the recently determined crystal structures of Escherichia coli E. coli  **MutS** , MutL and  **MutH**  to guide construction of 47 amino acid substitutions in these proteins and analyzed their behavior in mismatch repair and recombination in vitro and in vivo.  Ref: 12606120 DNA Repair Amst , 2003
- To investigate the effects on PV rates of mutations in genes for mismatch repair MMR, insertion deletion mutations of  **mutS** , mutL,  **mutH** , dam, polI, uvrD, mfd and recA were constructed in Hi strain Rd.  Ref: 11889052 EMBO J, 2002
- At a low dose, dP is much more mutagenic in mismatch repair defective  **mutH** , mutL, and  **mutS**  strains than in a wild type strain.  Ref: 12196386 Genetics, 2002
- Strand specificity and repair efficiency are largely independent of the GATC methylation state of the DNA and presence of the products of mismatch repair genes  **mutH** , mutL, and  **mutS** .  Ref: 12692132 J Biol Chem< MedlineTA>J Biol Chem, 2002
- **mutS**  and  **mutH**  are disrupted in mutants 3 11 and 12 23, and ssaQ is disrupted in mutant 17 21.  Ref: 12654850 Infect Immun, 2003
- The human mismatch repair hMMR system consists of members of three super families, namely  **MutH** , MutL and  **MutS** .  Ref: 12578652 Zhongguo Shi Yan Xue Ye Xue Za Zhi< MedlineTA>Zhongguo Shi Yan Xue Ye Xue Za Zhi, 2003
- **MutH** , MutL and  **MutS**  are essential components of the mismatch repair system in Escherichia coli.  Ref: 11934505 FEMS Microbiol Lett, 2002
- In vitro and in vivo studies of  **MutS** , MutL and  **MutH**  mutants correlation of mismatch repair and DNA recombination.  Ref: 12606120 DNA Repair Amst , 2003
